# 2142: A competency-based approach to redefining clinical research workforce quality and development

**DOI:** 10.1017/cts.2017.161

**Published:** 2018-05-10

**Authors:** Rebecca Namenek Brouwer, Denise Snyder

## Abstract

OBJECTIVES/SPECIFIC AIMS: Describe the process used to develop job descriptions and how this translates into consistent hiring practices. Describe how competencies are used to provide transparency into professional development opportunities. Discuss planned incorporation of competencies into efforts to train the clinical research workforce. METHODS/STUDY POPULATION: These processes were developed at Duke, an academic medical center with over 2000 active clinical research protocols and 300 new clinical trials per year. Over 1000 employees were evaluated for mapping into clinical research positions, with 685 mapping into new research positions (makeup of workforce to be depicted). RESULTS/ANTICIPATED RESULTS: Prior to this initiative, the clinical research workforce was not well-defined. Through the mapping process, employees were mapped from over 80 different positions into 10 (figure), resulting in a workforce that allows for visible career ladders and greater opportunity for development. As the initiative evolves and grows to include competency-driven performance evaluations, training modules, and assessments, we anticipate the ability to see the relationship between the competencies and high-quality clinical research support. DISCUSSION/SIGNIFICANCE OF IMPACT: The use of competencies in the context of workforce development is not new, yet in clinical research, they provide a much-needed framework for an ever-evolving profession. This comprehensive use of competencies throughout a workforce development initiative is key to ensuring strong support of high-quality clinical research.

